# Targeting Autophagy as a Strategy for Developing New Host-Directed Therapeutics Against Nontuberculous Mycobacteria

**DOI:** 10.3390/pathogens14050472

**Published:** 2025-05-13

**Authors:** Jia Wang, Sunhee Lee

**Affiliations:** Department of Microbiology and Immunology, University of Texas Medical Branch, Galveston, TX 77555, USA

**Keywords:** nontuberculous mycobacteria, autophagy, host-directed therapies, *Mycobacterium tuberculosis*, macrophages

## Abstract

Nontuberculous mycobacteria (NTMs) are increasingly being recognized as opportunistic pathogens in clinical practice because of their innate resistance to antimicrobial treatment and the widespread increase in multidrug-resistant strains on a global scale. NTMs pose a tremendous infection management challenge, especially in individuals with pre-existing lung conditions, as well as those who are immunocompromised. NTMs’ capability to evade or suppress the immune responses of their host is a key feature that makes them a cause of persistent chronic infection. Autophagy, an essential cellular defense mechanism that delivers and breaks down intracellular materials in lysosomes, protects the host from mycobacterial infection. Initial studies have revealed encouraging therapeutic strategies that augment endogenous autophagic mechanisms or block harmful host responses, thus having the potential to decrease intracellular mycobacterial infection, including that caused by multidrug-resistant strains. This review discusses how NTMs can evade autophagic mechanisms and considers the possibilities of using autophagy-inducing agents to develop novel therapeutic strategies to combat NTM infection.

## 1. Introduction

Nontuberculous mycobacteria (NTM) refers to mycobacterial species other than *Mycobacterium tuberculosis* complex and *M. leprae*, encompassing approximately 200 species, of which approximately 30 are pathogenic in humans [1]. NTMs are heterogeneous environmental bacteria ubiquitous in soil, water, dust, and environmental amoebae [2,3]. Infection with NTMs can cause pulmonary, extrapulmonary, or disseminated diseases [4]. While effects become visible in individuals with functional immune systems, those with underlying pulmonary conditions, like cystic fibrosis, bronchiectasis, and emphysema, and individuals with impaired immune defenses exhibit significantly increased susceptibility to non-tuberculous mycobacterial (NTM) lung disease [5,6,7,8,9].

Given the increased susceptibility of some populations to NTM infections, it is critical to study host cell processes like autophagy that are implicated in immune defense and clearance of pathogens. Autophagy is a critical and sophisticated cellular process preserved in numerous species. Autophagic mechanisms enable the breakdown of intracellular materials such as proteins, organelles, and exogenous substances. Further, autophagy is essential in maintaining cellular homeostasis by removing damaged organelles, misfolded proteins, and toxic aggregates, as well as by preventing cellular damage and stress [10,11,12,13]. Autophagy is also essential in the regulation of inflammation and in supporting the host’s immune system by breaking down components that may otherwise induce an overwhelming immune response [14].

Findings establish autophagy as being effective for the degradation of *M. tuberculosis*, for the improvement of antigen presentation, and the interaction of the innate and adaptive immune system, thus supporting the acquired immunity of the host [15,16,17]. *M. tuberculosis*, nevertheless, has evolved mechanisms to antagonize autophagic killing, promoting intracellular survival [18,19,20]. With these observations, heightened interest is focused on applying autophagy-targeting strategies for host-directed therapies (HDTs) to improve the treatment of tuberculosis [14]. Despite this, the function of autophagy in NTM infection is poorly understood. The exact mechanisms through which NTMs interact with and avoid the host autophagy machinery are not characterized [21]. In this review, we aim to discuss the molecular mechanisms taken up by NTMs to regulate autophagy. We will examine how such knowledge can be translated into developing HDTs for mycobacterial infection treatment.

## 2. NTM Overview and Pathogenic Spectrum

NTMs are aerobic, gram-positive, and non-motile bacilli with lipid-rich cell walls containing glycolipids, lipoproteins, and mycolic acids [22]. One of the distinguishing features of some NTM species is the presence of glycopeptidolipids (GPLs), which are absent in *M. tuberculosis* [23]. NTMs can be generally divided into rapidly growing mycobacteria (RGMs) and slowly growing mycobacteria (SGMs), depending on the growth observed on solid media. For example, RGMs, such as *M. abscessus*, *M. chelonae*, and *M. fortuitum*, require less than 7 days to produce visible growth, whereas SGMs, such as *M. avium* complex (MAC), usually require more than 2 weeks, even up to 12 weeks, to grow [24,25,26].

Pulmonary disease is the predominant NTM manifestation in adults, presenting with nonspecific symptoms such as chronic cough, dyspnea, fatigue, anorexia, and weight loss, along with radiological findings [4,5,9,22,27,28]. MAC and *M. abscessus* complex (MABC) are the leading causes, accounting for up to 85% of global NTM-PD cases [29]. Less frequent but clinically relevant species include *M. kansasii* and *M. xenopi* [30]. NTM pulmonary disease (NTM-PD) can be a progressive condition and may sometimes lead to mortality. Radiologically, MAC-PD presents in two dominant subtypes: nodular bronchiectasis (often seen in non-smoking elderly women) and fibrocavitary disease (more aggressive, frequently associated with smoking or chronic lung diseases) [31,32,33]. A third radiological pleuroparenchymal fibroelastosis (PPFE)-associated phenotype has also been reported and is associated with poor prognosis [34,35].

In addition to pulmonary disease, species like *M. abscessus*, *M. chelonae*, *M. fortuitum*, *M. marinum*, and *M. ulcerans* can cause skin, soft tissue, and bone infections [28,36,37,38]. These typically appear as chronic, non-healing lesions following trauma or surgery [7,39]. In children, the most common infection site is the lymph nodes, followed by the lungs and skin [28,40]. Immunocompromised individuals, including those with HIV/AIDS, cancer, or post-transplant immunosuppression, are especially susceptible to disseminated and often fatal infections [4,8].

## 3. NTM Disease Risk Factors

The global prevalence of NTM-PD has increased in recent decades, especially in high-income countries. In the United States, prevalence rose from 6.8 to 11.7 per 100,000 persons between 2008 and 2015, with considerable regional variation [41,42,43]. Risk factors include both environmental and host-related conditions [9,44,45]. Structural lung diseases such as bronchiectasis and COPD, mucociliary clearance defects like CF and primary ciliary dyskinesia, and immunosuppressive states are major contributors.

A distinct clinical presentation, known as Lady Windermere syndrome, primarily affects postmenopausal women with low BMI and impaired cough reflex. Genetic studies have associated this condition with MST1R mutations, which impair mucociliary clearance and increase susceptibility to chronic NTM colonization [46,47]. NTM infections are increasingly recognized in cystic fibrosis (CF), where impaired phagocytosis and abnormal mucociliary function, often due to CFTR mutations like F508del, foster infection risk [48,49]. Between 2010 and 2021, NTM infection prevalence in CF patients was 7.9%, with MABC and MAC being the most common pathogens [50]. A similar rise has been noted in bronchiectasis, with prevalence reaching approximately 10%, driven mainly by MAC [51]. These trends are likely multifactorial, involving aging populations, immunosuppressive therapies, enhanced diagnostic tools, and environmental changes [9,43].

Despite better recognition, the epidemiology of NTM-PD remains difficult to delineate due to species diversity, environmental ubiquity, and variable diagnostics [5,7,52]. Recent findings implicate the PARK2 gene, involved in autophagy, as a potential biomarker for susceptibility to NTM-PD.

## 4. Challenges in Therapy and the Need for Novel Strategies

Current treatment for NTM-PD involves prolonged multidrug antibiotic regimens, often exceeding one year and requiring culture negativity for 12 months to consider cessation. These regimens include injectables, drugs with significant adverse effects, and agents that usually interact with medications used for comorbidities, posing significant adherence challenges [29,53]. Five-year mortality for MAC-PD remains high, ranging from 10% to 48% [54]. MABC infections are complicated to treat due to intrinsic resistance and biofilm formation, which enhances adherence and immune evasion [55,56,57,58].

NTMs’ ability to survive within macrophages adds to treatment complexity by protecting the organisms from immune responses and antibiotics [59,60]. These factors contribute to elevated morbidity and mortality in susceptible populations such as the elderly and those with existing lung diseases [61,62].

Given these challenges, host-directed therapies (HDTs) are gaining attention as a complementary or alternative strategy. By targeting host pathways like autophagy, HDTs enhance pathogen elimination while reducing reliance on traditional antibiotics [63,64,65]. Unlike conventional drugs, HDTs may also eliminate dormant bacterial populations, making them a promising solution to combat antibiotic resistance [24,66,67,68]. Of relevance is the fact that autophagy modulation is a highly promising intracellular mechanism being explored for future therapeutic applications.

## 5. Types and Mechanisms of Autophagy

Autophagy is a highly conserved process through which eukaryotic cells degrade their cytoplasmic components via lysosomes to maintain cell survival and homeostasis [69]. This process can occur in different forms depending on how cargo is targeted and delivered to lysosome and membrane dynamics. Three canonical autophagy pathways have been described: microautophagy, chaperone-mediated autophagy, and macroautophagy [70,71].

Microautophagy involves lysosomal membrane invagination or deformation to take up cytoplasmic contents directly. In contrast, chaperone-mediated autophagy involves directly transporting cytosolic cargo proteins to lysosomes via the help of chaperone HSC70 and lysosomal membrane protein LAMP2A [71]. Macroautophagy, the most extensively studied form of autophagy, is characterized by forming a double-membraned structure called an autophagosome that engulfs cytoplasmic material and subsequently fuses with a lysosome for degradation [10,72]. Non-selective or selective macroautophagy can be activated under stress conditions such as starvation, hypoxia, or infections [73,74,75].

While autophagy can degrade substrates in bulk, selective autophagy employs specific receptors known as selective autophagy receptors (SARs) to target specific components for degradation [75]. In mammals, the most-studied soluble SARs are the sequestosome-1-like receptors (SLRs) p62, NBR1, NDP52, TAX1BP1, and Optineurin (OPTN) [14]. In selective autophagy, specific cargos are first tagged by ubiquitination, followed by recognition of the autophagy adaptor molecules for subsequent targeting to autophagosomes for degradation [76]. P62 recognizes several eat-me signals, including mono- and/or polyubiquitin, recognized by the ubiquitin-associated domain of p62 and/or NBR1 [77,78,79]. Another important eat-me signal used to label damaged lysosomes or bacteria-containing vacuoles consists of cytosolic lectins of the GAL family. GALs interact with β-galactosides, and the binding of GALs to intraluminal sugars is exposed when a ruptured membrane works as an eat-me signal [14,75].

Aside from the homeostatic functions of autophagy, one form of selective macroautophagy, named xenophagy, can capture and break down intracellular pathogens via lysosomal degradation to control infections [14]. Xenophagy involves the same steps as canonical autophagy, which are often delineated as initiation, elongation, substrate targeting, and maturation/lysosomal fusion (resulting in cargo degradation) [80].

### 5.1. Molecular Regulation of Autophagosome Formation

Autophagy is integral to innate immunity and is designed to kill intracellular organisms like mycobacteria by lysosomal degradation. During *M. tuberculosis* infection, autophagy supports phagosome maturation, enhancing antigen presentation and restricting the survival of bacteria within macrophages. However, many pathogenic mycobacteria have developed counterstrategies to block or modulate autophagic flux, such as blocking autophagosome–lysosome fusion and disrupting autophagy signaling pathways. The findings, primarily those of studies of *M. tuberculosis*, provide a framework to study the role of autophagy in the disease control of NTMs and to inform host-targeting therapy to restore or boost this immune mechanism [81].

There are three main protein complexes involved in regulating canonical autophagy: the ULK1 complex, the class III phosphatidylinositol 3-kinase (PI3KC3-C1) complex, and the conjugation machinery that consists of ATG5, ATG12, and ATG16L1. The process is typically initiated by stress or nutrient signal deprivation, followed by AMPK activation and inhibition of mTORC1 [82]. Autophagy regulatory pathways are highly conserved and offer multiple molecular nodes for therapeutic modulation. Drugs that activate AMPK or TFEB or inhibit mTORC1 exploit this machinery to enhance autophagic flux mechanisms increasingly relevant to host-directed therapy against intracellular pathogens, including NTMs.

Autophagy initiation begins with the formation of the ULK1 complex (ULK1/ULK2, ATG13, FIP200, ATG10), which translocates to the endoplasmic reticulum (ER) [83]. It then recruits the PI3KC3-C1 complex (Beclin 1, VPS34, VPS15, ATG14L1), generating phosphatidylinositol 3-phosphate (PI3P) to nucleate the phagophore [84]. PI3P recruits effector proteins like WIPI2 and DFCP1, which in turn assemble the ATG12–ATG5–ATG16L1 complex at the membrane [85].

The mechanism of phagophore elongation consists of two different ubiquitin-like conjugation processes: one that involves the ATG12–ATG5–ATG16L1 complex and another that relates to the lipidation of ATG8 family member LC3. LC3 precursor is processed to LC3-I by ATG4 and then lipidated by ATG7 and ATG3 to form LC3-II, which then becomes membrane-associated at the autophagosomal membrane [85]. LC3 plays an integral role in selective autophagy by binding to autophagy receptors that recognize intracellular cargo destined to be degraded. Autophagosome formation and subsequent fusion with lysosomes to form autolysosomes depend on the PI3KC3 complex II, which contains UVRAG instead of ATG14 [85,86,87]. The contents undergo degradation by lysosomal enzymes and are then recycled.

### 5.2. LC3-Associated Phagocytosis (LAP): A Non-Canonical Autophagy Pathway

LC3-associated phagocytosis (LAP) represents a distinct form of non-canonical autophagy that aids host defense [67]. Unlike classical autophagy, which engulfs cytoplasmic cargo, LAP specifically targets extracellular material. It is initiated by the recognition of pathogens by receptors on the surfaces of phagocytes, such as Toll-like receptors (TLRs), dendritic cell-associated C-type lectin-1 (dectin-1), and Fcγ receptors (FcγRs), followed by phagocytosis and internalized cargo within a single membrane phagosome [88].

Subsequently, the class III PI3-kinase complex, consisting of VPS34, Beclin-1, UVRAG, and Rubicon, is recruited and generates PI3P to decorate the compartment. The recruitment and assembly of the NADPH oxidase 2 (NOX2) complex triggers the production of reactive oxygen species (ROS). ROS production recruits the autophagic conjugation systems ATG7-ATG3 and ATG12–ATG5–ATG16L1, leading to the rapid lipidation of LC3-I to LC3-II and conjugation to PE on a single-membrane phagosome, forming LAPosome [87]. LC3-II decoration mediates the fusion of the LAPosomes with lysosomes, effectively eliminating engulfed pathogens.

## 6. NTM and Antimicrobial Responses of Macrophages and Autophagy Targeting Compounds Against NTM Infections

Xenophagy and the LAP pathways contribute to macrophages’ response to *M. tuberculosis* infection [89,90]. Autophagy confers protection against mycobacterial infection by reducing bacterial growth and inflammation [91,92]. High-virulent mycobacteria, such as *M. tuberculosis*, *M. bovis* BCG, and *M. kansasii*, induce markedly weaker autophagic responses in host macrophages than avirulent mycobacteria, including *M. smegmatis* [93]. This is partly because highly virulent mycobacteria evolved tactics to evade host defenses by blocking phagosome maturation or inhibiting stages of autophagy in infected host cells [94,95,96,97,98]. While conditional knockdown of the core autophagy component ATG5 in myeloid cells confers extreme susceptibility to *M. tuberculosis* in mice [91], knockout of ATG16L1 or ATG7 promoted the growth of *M. tuberculosis* and increased host susceptibility in mice to a lesser degree than that of ATG5-depleted mice [99]. In the absence of ATG5, ATG7, or ATG16L1, *M. tuberculosis* more frequently escapes from phagosomes in an ESX-1-dependent manner [99]. Manipulation of xenophagy by inhibiting the maturation of autophagosomes is well-studied in *M. tuberculosis*. *M. tuberculosis* has evolved advanced mechanisms to escape the autophagy machinery.

Beyond *M. tuberculosis*, NTM species such as *M. avium* and *M. abscessus* exploit host immune pathways to evade autophagic degradation. Unlike *M. tuberculosis*, which acts specifically to inhibit autophagy at the maturation stage of autophagosomes, several NTMs have shown a capacity to regulate host immune responses by influencing lipid metabolism, disrupting autophagic flux, and inhibiting antigen presentation [21,100,101]. The results of this study indicate that pharmacologic agents that can induce autophagy are increasingly considered to be a potential therapy to enhance the ability of the host to clear infection [102,103]. As resistance in mycobacterial strains continues to increase, there is growing interest in using autophagy to reinforce host defense mechanisms and achieve better treatment outcomes (Table 1).

### 6.1. Mycobacterium avium Complex (MAC) and Autophagy

MAC mainly consists of *M. avium*, *M. intracellular*, and *M. chimaera* species. The *M. avium* can be divided into four subspecies: *M. avium* subsp. *avium* (MAA), *M. avium* subsp. *Silvaticum* (MAS), *M. avium* subsp. *Paratuberculosis* (MAP) and *M. avium* subsp. *Hominissuis* (MAH) [136]. MAA and MAS are highly host-specific pathogens that primarily infect birds, while MAP is the causative agent of Johne’s disease in ruminants and wildlife. In contrast, *M. intracellular* and MAH are widely distributed environmental bacteria. The bacterium forms robust biofilms that allow it to colonize and persist in challenging environments, such as residential and commercial water systems. The most common route of MAC entry into the body that causes pulmonary disease is inhalation from the environment. In addition, MAC can also infect humans through the gastrointestinal tract or by direct inoculation from trauma or invasive medical interventions [137].

*M. avium* enters macrophages via the complement receptors CR1, CR3, CR4, and the mannose receptor [138]. Following the mechanism of phagocytosis, *M. avium* employs several strategies for subverting macrophage defense mechanisms and maximizing its intracellular longevity. Among the key mechanisms is the ability to survive in a non-acidified phagosome. The bacterium prevents the accumulation of proton-ATPases, which are responsible for acidifying endosomes and phagosomes, effectively halting phagosomal acidification [139,140]. Additionally, *M. avium* can also arrest the phagosome maturation process. The small-molecular-weight GTPases Rab5 functions in endocytosis from the plasma membrane and homotypic fusion between early endosomes. Rab7 regulates transport from the early endosome to the late endosome. *M. avium* phagosomes retain Rab5 but fail to acquire Rab7, which requires accessibility to iron, inhibiting the phagosome maturation process [141].

Beyond interfering with phagosome maturation, *M. avium* is highly resistant to ROS and nitric oxide (NO), allowing it to evade macrophage-killing [142]. The bacterium expresses superoxide dismutase enzymes that neutralize ROS, protecting against oxidative stress within macrophages and neutrophils [143,144]. Moreover, *M. avium* secretes MAV_4644, a dual-function protein with putative pore-forming function and ADP-ribosyltransferase activity, interacts with the host lysosomal peptidase cathepsin Z, and protects the early macrophage killing of *M. avium* by reactive nitrogen intermediaries [145]. In addition, *M. avium* also disrupts the processing of pro-cathepsin L to active forms, which influences antigen processing and presentation by MHC class II molecules in macrophages [146]. Several MAH genes can prevent phagosome maturation, inhibit phagosome–lysosome fusion, or make bacteria resistant to oxidative stress to promote intracellular survival [136].

Live and heat-inactivated *M. avium* strain 104 have both been shown to induce autophagy in macrophages, thus reducing intracellular viability. However, a fraction of *M. avium* persists despite autophagic activation [147], which suggests that while autophagy contributes to bacterial clearance, it does not eliminate the pathogen.

Microarray analysis of *M. avium*-infected THP-1 cells revealed a significant increase in MiR-125a-5p expression 24 h post-infection. Overexpression of MiR-125a-5p enhances autophagy and reduces intracellular *M. avium* survival by inhibiting its target STAT3 [148]. Additionally, cholesterol depletion in *M. avium*-infected macrophages resulted in phagolysosome-derived autophagy [149]. Following cholesterol replenishment, *M. avium* shifted from residing in double-membrane autophagolysosomes to immature phagosomes, its typical vacuolar niche, which suggests a subset of intracellular *M. avium* can evade and survive autophagy [149].

Beyond mammalian hosts, *M. avium* subspecies can survive within environmental amoebae. Encountering free-living amoebae has been found to provide several survival advantages to *M. avium*, most notably the acquisition of resistance to cell death by autophagy in both protozoan and mammalian macrophage environments [60]. The *M. avium* components responsible for suppressing or stimulating autophagy and the factors controlling these processes are unknown, thus presenting a key gap in the knowledge of *M. avium*’s intracellular means of survival.

### 6.2. Autophagy Targeting Therapeutics Against MAC Infection

There has been an inquiry into the possibility of targeting autophagy to improve the host’s capacity to control MAC infection. Nevertheless, evidence indicates that regulating autophagy could be a practical therapeutic approach. In particular, the autophagy inducer rapamycin suppresses MAP’s in vitro growth [150]. Previous work using a drug repurposing screen of a library of compounds that impact autophagy identified the antiarrhythmic drug amiodarone as reducing the intracellular load of *M. tuberculosis* in human cell culture [151]. Later, it was found that amiodarone also impaired the survival of *M. avium* in primary human macrophages by enhancing the autophagy response by increasing the activation of the significant autophagy regulator transcription factor EB (TFEB) [102]. Additionally, amiodarone also effectively reduced mycobacterial burden in vivo in the zebrafish embryo TB model [102], which suggested the possibility of exploiting autophagy as a target for HDT during NTM infection.

Lactoferricin, an antimicrobial peptide derived from the cleavage of the highly cationic N-terminal domain of the iron-binding protein lactoferrin, exhibits activity against *M. avium* strains of varying virulence. The bovine-derived lactoferricin peptide demonstrates superior antimicrobial activity compared to the human-derived form [152]. The D-enantiomer of bovine lactoferricin (D-LFcin17–30), which includes amino acids 17 to 30, is more resistant to proteolytic degradation and displays greater efficacy than the L-form [152]. Interestingly, the follow-up study reported the activation of lysosomal and autophagic pathways in macrophages by D-LFcin17–30, which can be crucial for its capacity to kill the intracellular *M. avium* clinical strain. Furthermore, D-LFcin17–30 also synergizes with ethambutol in inhibiting *M. avium* growth inside macrophages [133], indicating that autophagy induction is a viable HDT target against MAC infections.

Trehalose has been shown to induce xenophagy flux, which enables the killing of *M. avium* and *M. tuberculosis* during HIV co-infection by reversing the HIV-mediated autophagy block [117]. Another promising HDT candidate is alpha-1-antitrypsin (AAT). AAT infusion enhances primary human macrophage control of *M. intracellulare* infection by induction of phagosome–lysosome fusion and autophagy in AAT-deficient subjects [118]. These findings suggest that AAT replacement will not only help delay the progression of emphysema and bronchiectasis due to the deficiency but may also be a potential adjunctive HDT to the antibiotics to treat a concomitant NTM lung infection.

Mefloquine, an antimalarial agent, is active against MAC in vitro, in macrophages, or in mouse models [108,109]. Mefloquine also induces autophagy in neuroblastoma cells [110]. Induction of the autophagy mechanisms of the host by mefloquine may enhance its activity against MAC in vivo. Likewise, thioridazine, a typical phenothiazine antipsychotic drug, eradicates MAC both directly and intracellularly because of the entrapment of the drug within lysosomes [113,115,116]. Thioridazine increases moxifloxacin’s effectiveness, showing a rapid clearance of MAC in hollow-fiber models [116]. At the same time, thioridazine induces autophagy by enhancing the activity of AMP-activated protein kinase (AMPK) and the Wnt/β-catenin-p62 signaling pathway in glioma cells [112]. Thioridazine can also induce the autophagic response of the host as a way of fighting MAC infections.

Carvacrol, a phenolic monoterpenoid from several aromatic plants, has antimicrobial activity against MAP and rapidly growing mycobacteria (RGM), such as MABC [123,124]. There is variability in previous studies related to the autophagic function of carvacrol in different tissues. Significantly, carvacrol inhibits autophagy during adipogenic differentiation [125]. In addition, carvacrol enhances the resistance of HeLa cells to cisplatin by activating ERK1/2-mediated autophagy [126].

Nilotinib, a second-generation tyrosine kinase inhibitor used for treating chronic myeloid leukemia, can improve the ability of infected macrophages to eliminate both MAP and *M. bovis* [111]. Nilotinib promotes the autophagic degradation of *M. bovis* by inhibiting the PI3K/Akt/mTOR pathway via c-ABL and by activating the parkin protein, which subsequently enhances the ubiquitination of *M. bovis* in infected macrophages [111]. These findings indicate that autophagy is an active antimicrobial mechanism against *M. avium.*

### 6.3. Mycobacterium abscessus Complex (MABC) and Autophagy

MABC group consists of *M. abscessus* subsp. *abscessus*, *M. abscessus* subsp. *massiliense*, and *M. abscessus* subsp. *bolletii*. The role of autophagy in host defense against MABC infection may vary depending on the morphological type, including smooth or rough variants, strains, and infected cell types. Generally, the MABC rough variant induces more autophagy than the smooth variant, which may be due to a lack of GPLs on the rough variant that could mask the underlying phosphatidyl-myo-inositol mannosides (PIMs), the major non-peptidic antigens of the host innate and acquired immune response [153,154]. Compared to the MABC ATCC smooth strain, the clinical strain UC22 with rough morphology induced an elevated autophagy response. However, despite triggering autophagy, UC22 inhibits autophagosome–lysosome fusion (i.e., autophagic flux), thereby promoting its intracellular survival. The lipids of UC22 stimulated this high autophagy response, but whether they are responsible for inhibiting autophagy flux remained uncharacterized [155].

Azithromycin, identified as a front-runner drug for treating MABC-instigated infections, has shown the ability to interfere with the autophagosomal clearance mechanism by blocking lysosomal acidification. The dysfunction of the autophagic degradation pathway in primary human macrophages can increase the susceptibility of cystic fibrosis (CF) patients to infection by the MABC [127]. Follow-up research has invalidated the role of autophagy in MABC elimination by immune phagocytic cells, especially neutrophils. Research showed that neither azithromycin nor autophagy modulators affected the mycobactericidal activity of neutrophils [156]. These findings underscore the continued poor mechanistic understanding of the different MABC strains that use autophagy to evade the host’s defense.

### 6.4. Autophagy Targeting Therapeutics Against M. abscessus Infection

Autophagy modulation has emerged as a promising strategy for HDT in MABC infections. Several compounds have demonstrated efficacy in enhancing autophagic pathways to improve bacterial clearance and reduce inflammation. Resveratrol, a Sirtuin 3 (SIRT3) agonist, is a mitochondrial protein deacetylase with antioxidant, anti-inflammatory, and immunomodulatory properties [157]. It has been shown to reduce MABC growth and attenuate inflammation and tissue damage in mice or zebrafish infected with MABC [158]. SIRT3 has been linked to antibacterial autophagic mechanisms acting on *M. tuberculosis* by acting as a mediator for the nuclear receptor peroxisome proliferator-activated receptor α (PPARα), which is a key transcription factor in maintaining mitochondrial homeostasis and enhancing autophagy in the course of an infection [159]. The rapid metabolism and poor bioavailability of resveratrol, however, are significant challenges to its potential practical application in a therapeutic setting. V46, a resveratrol analog, has been developed to overcome these challenges and exhibits potent antimicrobial activity against *M. abscessus* rough and smooth variants in macrophages, mice, and zebrafish infected with MABC. Interestingly, V46 induces autophagy in MABC-infected host cells via a SIRT3-independent mechanism. It results in nuclear translocation of the master regulator of lysosomal biogenesis and autophagy, TFEB, via inhibition of the Akt-mTOR pathway but activation of the AMPK pathway [107].

Activation of peroxisome proliferator-activated receptor alpha (PPARα) is a mechanism that induces autophagy by promoting autophagy-related genes (ATGs) independently of the mTORC1 pathway [160]. Evidence suggests that PPARα agonists can induce autophagy, increase phagosome maturation, and trigger protective immune responses to Bacillus Calmette–Guérin (BCG) and *M. tuberculosis*. It is achieved through the modulation and nuclear translocation of transcription factor EB (TFEB) [161]. Gemfibrozil, a lipid-moderating drug used clinically and as a known PPARα agonist, has shown profound effects by decreasing bacterial burden and alleviating subsequent pathological inflammation associated with mycobacterial abscesses in mouse models [106]. The activation of TFEB is key for the antimicrobial response; thus, activation of autophagy through PPARα-TFEB could increase gemfibrozil’s effectiveness in treating MABC infection. Additionally, resveratrol and gemfibrozil were shown to act as lysosomal activators in MABC infection [106,158].

The modulation of host metabolic pathways has been proposed as a possible approach for treating MABC infection. Studies show that the pyruvate dehydrogenase kinase (PDK)-inhibiting action of dichloroacetate disrupts the increased glycolytic metabolism of *M. abscessus* subsp. *massiliense* infection. Modulation of this metabolism brings the host immune response back into balance and upregulates the phosphorylation of AMPKα1, which further activates autophagy and effectively restricts the intracellular growth of *M. massiliense* [121].

Certain antimycobacterial antibiotics have a bifunctional mode of action that affects both the mycobacterial pathogens and the host immune response. Rufomycin is one such antibiotic that acts selectively on ClpC1, the subunit of the caseinolytic protease complex that is critical in the extracellular growth and intracellular survival of *M. tuberculosis* and *M. abscessus* [128,129,162]. Rufomycin also induces autophagy and enhances lysosomal gene expression through the nuclear translocation of TFEB, enhancing the clearance of mycobacteria.

In addition, recent studies point toward the central role of CFTR dysfunction in the dysregulation of autophagic processes. CFTR is not only an ion channel but also a central regulator in the system of proteostasis, having a significant impact on a wide range of cell functions, including autophagy [163]. Dysfunctional CFTR increases reactive oxygen species (ROS) and tissue transglutaminase (TG2), leading to the cross-linking and trapping of Beclin 1. This dislodges the PI3KC3 platform from the endoplasmic reticulum, thus inhibiting autophagy initiation. Consequently, the autophagy substrate SQSTM1/p62 accumulates, trapping misfolded F508del-CFTR and key anti-inflammatory proteins such as PPARγ and IκBα in intracellular aggresomes, perpetuating a pro-inflammatory environment in cystic fibrosis airways [164].

Cysteamine, an aminothiol product of coenzyme A metabolism used for treating cystinosis, stimulates autophagy in cells with the F508del mutation, helping to stabilize CFTR at the plasma membrane [165]. Cysteamine and its analog cystamine, two TG2 inhibitors, effectively reduce *MABC* loads of both rough and smooth variants and decrease the size of human ex vivo granuloma-like structures. Furthermore, when combined with amikacin, enhanced anti-MABC effects were observed [105].

The second lipid messenger, phosphatidylinositol-5-phosphate (PI5P), which plays a role in phagolysosome biogenesis, has been explored as a therapeutic strategy against MABC. PI5P is delivered to infected macrophages via apoptotic body-like liposomes (ABLs), where it promotes intracellular bacterial killing through ROS production and phagosome acidification, particularly in macrophages derived from CF patients. ABL/PI5P treatment alone reduces bacterial burden and leukocyte recruitment in CF mice infected with MABC. ABL/PI5P combined with amikacin considerably enhances MABC clearance and increases anti-inflammatory effects in vivo and in vitro [104].

Tetracycline has been used as part of combination regimens for salvage therapy of MABC infections. Tetracycline induces autophagy through inhibition of the activity of mTOR [130]. A new derivative of tetracycline, omadacycline, has also exhibited greater tolerance and is suitable for oral use, besides showing efficacy against clinical strains of MABC, including drug-resistant strains [131,132,166,167,168]. In addition, in a murine model of MABC pulmonary infection, omadacycline was very potent [167,168]. However, the exact mechanism of how autophagy mediates in vivo clearance of MABC is unclear.

### 6.5. M. marinum and Autophagy

*M. marinum*, a slow-growing NTM closely related to *M. tuberculosis*, is often used as a model organism to study mycobacterial pathogenesis, particularly for tuberculosis-like diseases [169,170,171,172]. The organism usually grows at temperatures between 25 and 35 °C, and it is known to produce yellow pigments when exposed to light (photochromogenic). *M. marinum*, the causative agent of aquarium granuloma, has been isolated from human hosts. Aquarium granuloma is present in developing nodular or ulcerative lesions on the extremities. A delayed diagnosis can lead to progressive invasion of the tissues, synovial membrane, bursae, and bones in roughly one-third of cases [173]. Dissemination is rare but can happen in immunocompromised individuals [174,175].

The pathogen’s capacity to infect model organisms such as zebrafish (*Danio rerio*), *Dictyostelium discoideum*, and *Drosophila melanogaster* has offered valuable insights into host–pathogen interactions, particularly highlighting the role of autophagy in infection control [176,177,178,179,180]. The zebrafish model is especially valuable as it allows for real-time, in vivo visualization of infection dynamics using GFP-LC3 autophagy reporters.

Autophagy is a critical host defense mechanism against *M. marinum*, mainly mediated by the induction of autophagosome formation by the ESX-1 secretion system [181]. However, the bacterium can block autophagic flux, thus evading xenophagic degradation during infection [182]. Microglia suppress the bacterial growth of zebrafish via the autophagic process [183]. In addition, the pharmacological induction of autophagy by rapamycin results in the reduced viability of bacteria [182,184,185,186]. The advanced microscopic methods show that LC3 has a temporary and dynamic association with *M. marinum*, which generally results in extensive vesicle formation inside leukocytes. This feature indicates that the autophagic pathway has a selective mechanism [186,187].

In addition to zebrafish, *D. discoideum*, known for its phagocytosis abilities, sheds light on operating the Endosomal Sorting Complex Required for Transport (ESCRT) and the autophagic pathway in the context of responses to damaged membranes. The evolutionary conservation of these processes emphasizes their protective functions in host defense mechanisms [188,189]. Transcriptomic signatures show that infection by *M. marinum* causes the induction of ESCRT and autophagy-associated genes, which implies the presence of a specialized pathway for the repair of damaged membranes [177]. In *D. melanogaster*, autophagy, supported by ATG2, is responsible for suppressing bacterial growth within phagocytic cells and organizing lipid droplets [190]. Gene function studies have revealed ATG2 and ATG7 as critical autophagy regulators for *M. marinum* infection in Drosophila [18,176,190]. Further, ATG2 is characterized by other autophagic processes by being pathogen-specific toward bacterial pathogens. Significantly, the function of autophagy potentiates the effectiveness of the antibiotics, thus showing promise of a combinatorial therapeutic approach for the future treatment of mycobacterial infection [18].

Selective autophagy receptors, including OPTN and p62, play a pivotal role in the degradation of ubiquitinated *M. marinum*. Experiments using zebrafish models have shown that the absence of either OPTN or p62 leads to reduced autophagic targeting and heightened susceptibility to infection, while their overexpression promotes bacterial clearance [191]. Both OPTN and p62 also exhibit compensatory functions; however, the lack of DRAM1 highlights their overlapping yet discrete functions in host defense mechanisms. DRAM1 is critical in the recruitment of LC3 to *mycobacterium*-containing vesicles, which are implicated in the control of lysosomal degradation [192,193]. In dram1-deficient zebrafish, defective phagosomal maturation combined with increased bacterial load led to pyroptosis of the cell, thus further highlighting the critical role of dram1 in preventing macrophage apoptosis [191,192,194].

Aside from autophagy, membrane repair is also enabled with the assistance of the ESCRT machinery. ESCRT components VPS4, TSG101, and ALIX are assembled at the damaged membrane sites and act in conjunction with autophagy to preserve the membrane integrity [195,196,197,198]. In *D. discoideum*, the TRAF-like E3 ubiquitin ligase TrafE coordinates the interaction of the ESCRT pathway with autophagy, both of which are important in processes including xenophagy and membrane repair [189]. However, the autophagy and ESCRT pathways function separately at membrane damage sites, emphasizing their distinct yet complementary roles [188].

Notably, *M. marinum* employs multiple mechanisms to evade host autophagy. The ESX-1 secretion system disrupts the *Mycobacterium*-containing vacuole (MCV), which triggers autophagy gene expression while blocking autophagic flux to create a protective niche for intracellular survival [183]. Additionally, the phosphoribosyltransferase coded by the *mimG* gene is important for repressing autophagic and oxidative stress responses of the macrophages, which ultimately increases bacterial viability [199]. Once the bacteria escape from host cells, autophagy and ESCRT machinery components such as ATG8 become recruited to seal host membranes, allowing the non-lytic release of bacteria [21,196,200,201,202,203]. In the context of macrophages, *M. marinum*, being cytosolic, is rapidly ubiquitin-tagged and targeted to the LAMP-1^+^ vacuoles with an autophagy-independent mechanism, indicating the utilization of other clearance pathways [204].

Additionally, the metabolic processes of the host primarily affect the outcomes of infection events. In macrophages, for instance, *M. marinum* infection induces aerobic glycolysis, which supports bacterial growth. In contrast, the treatment with 2-deoxyglucose to suppress glycolysis reduces bacterial growth by stimulating autophagy while simultaneously inhibiting phagocytosis [16,21,205,206]. This shows a possible therapeutic approach by boosting the defense processes of the host.

### 6.6. Autophagy Targeting Therapeutics Against M. marinum Infection

Given the critical role played by autophagy in the management of *M. marinum* infections, several therapeutic methods have been investigated to boost this cellular process. Tamoxifen, which is a drug used in treating breast cancer, induces autophagic clearance of mycobacteria in both cultured macrophages and zebrafish models and reduces bacterial viability [114,120]. Of interest is that this specific process takes place through a mechanism that circumvents estrogen receptors, mainly stimulating lysosomal function and enabling the clearance of bacterial pathogens. In addition, it is also of interest that the pharmaceutical compound amiodarone, used to treat arrhythmias, induces autophagy by the transcription factor EB (TFEB), which leads to clearance of *M. marinum* and *M. avium* by autophagic mechanisms mediated by macrophages [102]. Its efficacy in zebrafish models further supports its potential as an HDT. Another anticancer drug, degarelix, reduces the survival of *M. marinum* and alleviates granuloma formation in zebrafish, showing effects comparable to those induced by rifampicin [119]. Degarelix’s mechanism promotes autophagy initiation via PI3 kinase activation, though the effects on later autophagic stages remain unclear.

Other promising autophagy-enhancing compounds include ohmyungsamycins (OMS), cyclic peptides derived from natural sources, which activate autophagy through AMPK signaling and promote bacterial clearance in *Drosophila* models of *M. marinum* infection [134]. OMS also suppresses inflammatory reactions, thus inducing beneficial effects on the elimination of bacteria and the modulation of the inflammatory response. Thiostrepton (TSR), a thiopeptide antibiotic defined by the presence of quinaldic acid, is active by a dual mechanism. Besides inhibiting ribosomal function in bacterial cells, it also induces autophagy in host cells due to endoplasmic reticulum stress. Such a secondary cell process intensifies host cell defense against *M. marinum*, thus highlighting the antibiotic’s potential as an immunomodulator in the host immune system [135].

### 6.7. M. ulcerans and Autophagy

Buruli ulcer (BU) is a chronic, necrotizing infection affecting dermal and subcutaneous adipose tissues. *M. ulcerans* is the causative organism of BU, with autophagy being a critical protective mechanism against *M. ulcerans* [207,208,209,210]. Mycolactone is the principal molecule responsible for the pathogenicity of *M. ulcerans*, with immune evasion and facilitation of tissue necrosis being the mechanism [209,210,211,212]. Mycolactone is responsible for its effects by interacting with the SEC61 translocon, which prevents protein translation into the endoplasmic reticulum (ER). Disruption of this translational process results in subsequent cytosolic translation and the integrated stress response (ISR). Activation of this pathway, in turn, enhances the process of selective autophagy, demonstrated by the increased levels of LC3B-II, ubiquitin, and SQSTM1/p62, thus protecting the cell from the harmful effects of protein deposition [213,214,215]. However, continuous activation of such stress responses, in particular through the activity of ATF4 (Activating Transcription Factor 4) and CHOP (C/EBP Homologous Protein) transcription factors, can trigger cellular impairment through excessive induction of autophagy and apoptosis [216].

Experimental systems have shown that the induced production of mycolactone re-establishes the pathogenicity of *M. ulcerans* within the in vitro and in vivo settings, highlighting its role in the modulation of autophagy and virulence promotion [214,217,218]. Genetic studies show that specific variants in autophagy-related genes influence the susceptibility of the host and disease progression. For example, PARK2 and NOD2 variations are linked with increased susceptibility to severe forms of BU, while the ATG16L1 rs2241880 variant is protective against ulcer development [219]. Additionally, a genome-wide association study identified two long non-coding RNAs and confirmed the protective effect of the *ATG16L1* variant, reinforcing the significance of autophagy in disease development [220,221]. These studies underscore the dual role of autophagy in *M. ulcerans* infection, acting as both a protective mechanism against cytotoxic stress and a contributor to disease progression when dysregulated, with host genetic factors significantly influencing clinical outcomes.

### 6.8. Autophagy Targeting Therapeutics Against M. ulcerans Infection

Given the role of autophagy in *M. ulcerans* infection, several autophagy-modulating compounds have been investigated for their therapeutic potential. Statins, including atorvastatin, fluvastatin, and simvastatin, exhibit antimicrobial activity against *M. ulcerans*, with fluvastatin being the most effective. Simvastatin activates autophagy via the AMPK-mTORC1-TFEB axis in macrophages [222]. The synergistic interaction of antibiotics like rifampicin and streptomycin augments the inhibition of bacterial growth, suggesting their use as adjunct therapies [122]. Protein Kinase R (PKR) can be a key regulator of immune response to *M. ulcerans* since mycolactone elicits autophagy by triggering the EIF2α-ATF4-CHOP pathway, with PKR likely enabling the phosphorylation of EIF2α [216].

The immunoenhancing effects of the tyrosine kinase inhibitor Imatinib mesylate are achieved by enhancing autophagy and inhibiting bacterial growth in host cells. The immunomodulatory effects of the drug are mediated by a dual mechanism involving autophagy induction and inhibition of bacterial growth in host cells. Imatinib-sensitive tyrosine kinases account for a significant molecular mechanism in mediating host cell invasion by mycobacteria and promoting subsequent macrophage colonization by the pathogens; both steps are crucial in the progression of mycobacterial infection [223]. Treatment by the kinase inhibitor imatinib reduces the bacterial load and delays the onset of granulomatous inflammation, thus providing a viable mechanism of host-targeting therapy for *M. ulcerans*. This mechanism is predicted to be less susceptible to resistance than conventional antibiotics. It also showed a synergistic result when combined with traditional antituberculosis treatments, highlighting the therapeutic potential of this mechanism [114,223,224,225]. Further studies will be needed to support these mechanisms and optimize their therapeutic application.

## 7. Conclusions and Future Perspectives on Autophagy-Targeted Therapies for NTM Infections

NTM infections pose significant clinical challenges due to their intrinsic antibiotic resistance, capacity to form biofilms, and capability to evade host immune defenses. Among host-targeting strategies, modulation of autophagy, including classical autophagy, xenophagy, and LAP, has proven to be an effective means to boost and optimize host intracellular defenses. Cysteamine, amiodarone, gemfibrozil, and resveratrol derivatives have emerged as effective compounds in inhibiting intracellular NTM load by activating pivotal autophagy regulators, i.e., AMPK, mTOR, and TFEB. Such drugs may be used as adjuncts to traditional antimicrobial treatments and hold significant promise in overcoming resistance by enhancing host defenses.

HDTs and antibiotics target fundamentally different aspects of infection, with HDTs acting on host immunity and antibiotics acting directly on bacterial components. This mechanistic divergence creates an opportunity for synergistic therapeutic outcomes, including reduced treatment duration, lower drug doses, and improved safety profiles. Autophagy-inducing HDTs such as carbamazepine and rapamycin have demonstrated the ability to enhance the activity of antibiotics and significantly enhance the clearance of both *M. tuberculosis* and NTMs [226]. Furthermore, recent studies highlight the effectiveness of combining HDTs with antibiotics, such as lactoferricin with ethambutol and ABL/PI5P with amikacin, for treating NTM infections, underscoring the potential of rationally designed combination regimens [104,133]. These dual-acting therapies could help overcome bacterial persistence and resistance while maintaining tolerability. However, the field still faces critical gaps. Despite promising outcomes, most studies limit their focus to a single HDT combined with antibiotics, overlooking the possibility that multiple HDTs with complementary modes of action could produce enhanced therapeutic outcomes.

Although NTM-specific HDT developments are promising, the therapeutic exploitation of autophagy-modulating compounds remains in its early stages. A significant limitation is the incomplete understanding of how different NTM species interact with autophagic pathways. While autophagy is broadly recognized as a host immune effector mechanism, the molecular mechanisms underlying NTM-autophagy interactions are still poorly defined. Most mechanistic insights are derived from *M. tuberculosis* studies, yet NTMs such as *M. avium*, *M. abscessus*, and *M. marinum* show species-specific responses to autophagy, reflecting divergent genetic and pathogenic traits. These organisms may block phagosome maturation, alter host lipid metabolism, or inhibit autophagic flux mechanisms that vary widely and remain undercharacterized. The complexity of this challenge is compounded by the multitasking interactions between autophagy and other host cellular processes, like inflammatory signaling and phagosomal trafficking. As such, attempts to identify therapy targets or predict implications of modulation of autophagy in the context of NTM infection pose considerable challenges. From a translational perspective, the development of treatment aimed at modulating autophagy and targeting the host is hindered by several reasons: lack of drug susceptibility breakpoints for multiple species, heavy reliance on studies used to derive these data on tuberculosis, limited preclinical testing of drugs in validated animal models, and limited understanding of long-term safety profiles.

Pharmacologic agents modulating autophagy hold significant potential to improve outcomes in drug-resistant and chronic NTM infections. Existing studies are increasingly applying the tools of systems biology, genetic screening, and precision medicine to inform the rational development of combination therapy regimens. Future studies should focus on elucidating species-specific regulatory networks, determining conserved mechanisms of autophagy evasion, and critically evaluating candidate compounds in clinically appropriate models. In addition, investigating interactions between autophagy inducers and conventional antimicrobials is an area of study that is relatively uncharted and potentially rich in reward. The development of these approaches will be crucial to fully realize autophagy’s therapeutic potential as a tool to counteract the rising global burden of NTM disease.

## Figures and Tables

**Table 1:** Therapeutic agents modulating autophagy: host-directed therapies and antibiotics against NTMs.

Therapeutic Agent	Original Drug Use/Classification	Mechanism of Action	Target NTMs	Reference
HDTs				
Apoptotic body-like liposomes loaded with phosphatidylinositol 5-phosphate	Experimental nanocarrier system	Stimulate phagolysosome biogenesis and enhance intracellular bacterial killing	*M. abscessus*	[104]
Cysteamine	FDA-approved for nephropathic cystinosis	Promotes autophagy, stabilizes CFTR function, and inhibits tissue transglutaminase	*M. abscessus*	[105]
Gemfibrozil	Lipid-lowering agent (fibrate class)	Induces autophagy via PPARα-TFEB pathway activation	*M. abscessus*	[106]
Resveratrol and V46	Resveratrol: Natural polyphenol (grapes, berries) V46: SIRT3 activator (experimental)	Activate SIRT3 and stimulate autophagy through AMPK signaling	*M. abscessus*	[107]
Amiodarone	Class III antiarrhythmic drug	Enhances autophagy through transcription factor EB (TFEB) activation	*M. avium* *M. marinum*	[102]
Mefloquine	Antimalarial drug (quinoline derivative)	Strongly induces autophagosome formation, especially in neuroblastoma cells	*M. avium* complex	[108,109,110]
Nilotinib	Tyrosine kinase inhibitor for chronic myeloid leukemia (CML)	Inhibits PI3K/Akt/mTOR via c-ABL; activates parkin-mediated autophagy	*M. avium*	[111]
Thioridazine	Typical antipsychotic (phenothiazine class)	Stimulates autophagy by increasing AMPK activity	*M. avium*	[112,113,114,115,116]
Trehalose	Naturally occurring disaccharide sugar	Induces xenophagy flux and reverses HIV-induced autophagy blockade	*M. avium*	[117]
Alpha-1-antitrypsin	Protein replacement therapy	Promotes autophagy and enhances phagosome–lysosome fusion	*M. intracellulare*	[118]
Degarelix	GnRH receptor antagonist for advanced prostate cancer	Triggers autophagy initiation via PI3K activation and lowers bacterial survival	*M. marinum*	[119]
Tamoxifen	Selective estrogen receptor modulator (SERM) for breast cancer	Stimulates lysosomal activity and promotes autophagy	*M. marinum*	[120]
Dichloroacetate	Investigational drug for metabolic disorders and cancer	Reprograms metabolism via AMPK activation, leading to autophagy induction	*M. massiliense*	[121]
Statins	HMG-CoA reductase inhibitors for hypercholesterolemia	Promote autophagy and synergize with antibiotics to improve efficacy	*M. ulcerans*	[122]
Carvacrol	Natural monoterpenoid phenol found in oregano and thyme	Inhibits MEK/mTOR signaling to promote autophagy; suppresses autophagy during adipogenic differentiation	*M. abscessus*, *M. chelonae*, *M. fortuitum*, *M. mucogenicum*, *M. avium*	[123,124,125,126]
Antibiotics				
Azithromycin	Macrolide antibiotics used to treat respiratory tract and intracellular infections	Inhibits lysosomal acidification, impairing autophagosome degradation; exhibits direct antimycobacterial activity	*M. abscessus*	[127]
Rufomycin	Ansamycin antibiotic; under investigation for antimycobacterial activity	Binds ClpC1 protease, activates TFEB, and upregulates autophagy-related gene expression	*M. abscessus*	[128,129]
Tetracycline	Broad-spectrum bacteriostatic antibiotic (inhibits protein synthesis)	Inhibits mTOR signaling, thereby activating autophagy	*M. abscessus*, *M. chelonae*, *M. fortuitum*	[130,131,132]
Lactoferricin (D-LFcin17–30	Antimicrobial peptide derived from bovine lactoferrin	Activates lysosomal pathways and promotes autophagy	*M. avium* complex	[133]
Ohmyungsamycins	Cyclic peptides from marine *Streptomyces* species with antimicrobial properties	Induce autophagy through activation of AMPK signaling	*M. marinum*	[134]
Thiostrepton (TSR)	Natural thiopeptide antibiotic with both antimicrobial and antitumor properties	Induces ER stress, leading to activation of autophagy	*M. marinum*	[135]
